# Factors influencing the implementation of chronic care models: A systematic literature review

**DOI:** 10.1186/s12875-015-0319-5

**Published:** 2015-08-19

**Authors:** Carol Davy, Jonathan Bleasel, Hueiming Liu, Maria Tchan, Sharon Ponniah, Alex Brown

**Affiliations:** 1 South Australian Health & Medical Research Institute, Adelaide, South Australia Australia; 2 The George Institute for Global Health, Camperdown, New South Wales Australia

## Abstract

**Background:**

The increasing prevalence of chronic disease faced by both developed and developing countries is of considerable concern to a number of international organisations. Many of the interventions to address this concern within primary healthcare settings are based on the chronic care model (CCM). The implementation of complex interventions such as CCMs requires careful consideration and planning. Success depends on a number of factors at the healthcare provider, team, organisation and system levels.

**Methods:**

The aim of this systematic review was to systematically examine the scientific literature in order to understand the facilitators and barriers to implementing CCMs within a primary healthcare setting. This review focused on both quantitative and qualitative studies which included patients with chronic disease (cardiovascular disease, chronic kidney disease, chronic respiratory disease, type 2 diabetes mellitus, depression and HIV/AIDS) receiving care in primary healthcare settings, as well as primary healthcare providers such as doctors, nurses and administrators. Papers were limited to those published in English between 1998 and 2013.

**Results:**

The search returned 3492 articles. The majority of these studies were subsequently excluded based on their title or abstract because they clearly did not meet the inclusion criteria for this review. A total of 226 full text articles were obtained and a further 188 were excluded as they did not meet the criteria. Thirty eight published peer-reviewed articles were ultimately included in this review. Five primary themes emerged. In addition to ensuring appropriate resources to support implementation and sustainability, the acceptability of the intervention for both patients and healthcare providers contributed to the success of the intervention. There was also a need to prepare healthcare providers for the implementation of a CCM, and to support patients as the way in which they receive care changes.

**Conclusion:**

This systematic review demonstrated the importance of considering human factors including the influence that different stakeholders have on the success or otherwise of the implementing a CCM.

**Electronic supplementary material:**

The online version of this article (doi:10.1186/s12875-015-0319-5) contains supplementary material, which is available to authorized users.

## Background

The increasing prevalence of chronic disease faced by both developed and developing countries is of considerable concern to a number of international organisations [1, 2]. Many of the interventions to address this concern within primary healthcare settings are based on the chronic care model (CCM) which was first developed by MacColl Institute for Healthcare Innovation at Group Health Cooperative in the early 1990s [3–5]. The elements included in this original model focused on mobilising community resources, promoting high quality care, enabling patient self‐management, implementing care consistent with evidence and patient preferences, effectively using patient/population data, cultural competence, care coordination, and health promotion [6].

The implementation of complex interventions such as CCMs requires careful consideration and planning. Success depends on a number of factors at the healthcare provider, team, organisation and system levels [7]. Implementation strategies should also take into account contextual factors [8]. As a result, primary healthcare services need to consider the range of interacting factors at many different levels and consider the possibility that multiple often interacting factors will largely determine whether a CCM is implemented and whether this intervention succeeds in improving health outcomes for people living with chronic disease [9].

A vast number of theories have been developed to inform the implementation of complex healthcare interventions [10]. Process theories focus on the activities and organisation of the change process, stage-of-change theories consider how the steps taken to implement the change differ according to the healthcare providers involved and impact theories describe how the intervention will facilitate change. There are theories that focus on individuals within the change process including cognitive, educational and motivational theories. There are also theories that relate to social interaction encompassing communication, social learning, social networking, team effectiveness, professional development and leadership theories. Finally, there are theories at an organisational level including integrated care and quality management, both of which underpin the development and implementation of CCMs [9].

A number of systematic literature reviews have already considered the effectiveness of CCMs [11–17]. None, however, have specifically focused on what impedes or promotes the successful implementation of CCMs. This systematic literature review goes some way to addressing this gap by identifying the facilitators and barriers to implementing CCMs within primary healthcare settings, from the perspective of both patients’ and healthcare providers’. The intention is that the outcomes from this review will assist both policy makers and practitioners working within a primary healthcare setting, to implement CCMs.

### Objectives

The specific purpose of this review was to systematically examine the scientific literature in order to understand the facilitators and barriers to implementing CCMs within a primary healthcare setting from the perspective of healthcare providers and patients. The question asked by this review was:

What attitudes, beliefs, expectations, understandings, perceptions, experiences, resources and knowledge according to healthcare providers and patients support (facilitators) or inhibit (barriers) the implementation of CCMs within a primary healthcare setting?

## Method of the review

A three-step search strategy was used in this review. An initial limited search of MEDLINE and CINAHL was undertaken followed by analysis of the text words contained in the title and abstract, and of the index terms used to describe article. A second keywords and index term search was then undertaken across Embase, Informit Online, PsycINFO, Scopus, and Web of Science. Duplications were then identified and the most complete record retained for subsequent review on inclusion criteria. Additional file 1 provides an example of the Medline search strategy.

### Inclusion criteria

#### Population and context

This review considered studies that focused on patients with one or more of the more prevlant major chronic diseases as defined by the World Health Organisation - cardiovascular disease, chronic kidney disease, chronic respiratory disease, type 2 diabetes mellitus and depression [18, 19] - receiving care in primary healthcare settings, as well as all primary healthcare providers such as doctors, nurses and administrators.

Primary healthcare is generally defined as first-contact, accessible, continued, comprehensive and coordinated healthcare provided by a single practitioner (e.g. GP, nurse practitioner) or a multidisciplinary team of professionals in a community practice. For the purposes of this review however, primary healthcare is first-contact, accessible, continued, comprehensive and coordinated care. First-contact care is accessible at the time of need; ongoing care focuses on the long-term health of a person rather than the short duration of the disease comprehensive care is a range of services appropriate to the common problems in the respective population and coordination is the role by which primary care acts to coordinate other specialists that the patient may need [20]. Primary healthcare also includes primary care settings that have only one health professional, i.e. a general practitioner (GP).

#### Phenomena of interest/intervention

The phenomena of interest were the attitudes, beliefs, expectations, understandings, perceptions, experiences, resources and knowledge of healthcare providers and patients about what supports (facilitators) or inhibits (barriers) the implementation of CCMs within a primary healthcare setting. To be included studies must have also referred to a CCM which included at least two of the following elements:

1. Facilitated community support **(CS)** to meet the needs of patients
2. Facilitated unpaid/informal family support **(FS)** to meet the needs of patients
3. Enhanced health care professional case management **(CM)** support to meet the needs of patients
4. Self-management support **(SMS)** to meet the needs of patients
5. Health organisational change **(OC)** to meet the needs of health-care providers
6. Delivery system design **(DSD)** to meet the needs of health-care providers
7. Decision support **(DS)** to meet the needs of health-care providers
8. Clinical information systems **(CIS)** to meet the needs of health-care providers

#### Outcome

Finally, this review only considered studies that included attitudes, beliefs, expectations, understandings, perceptions, experiences, resources and knowledge according to healthcare providers support (facilitators) or inhibit (barriers) the implementation of CCMs.

### Types of studies

This review focused on both qualitative and quantitative studies (e.g. randomised and non-randomised control trials, cross-sectional and cohort studies, case studies and case series). Papers were limited to those published in English between 1998 and 2013.

### Data collection

Data was extracted from primary studies and included in the review using a set of pre-defined tables. The extracted data included specific details about the chronic care model, populations, study methods and outcomes of significance to the review questions and objectives. Extracted data included:

- Study type
- Chronic disease
- Study setting (country and region)
- Chronic care elements

These data on the included studies are presented in an additional file [see Additional file 2].

### Critical appraisal

Two reviewers independently assessed the quality of the papers prior to inclusion in this review. The Cochrane Handbook for Systematic Reviews of Interventions was used to assess bias for randomised and non-randomised control trials, cross-sectional and cohort studies [21, 22]. The Joanna Briggs critical appraisal tool was used to measure the quality of case studies and case series [23]. As the objective of this review was to facilitators and barriers to implementing CCMs, studies were not excluded based on these critical appraisals.

### Data extraction

Data was extracted where possible by themes identified by the authors of each study. Where themes were not identified within the study, findings were extracted from the narrative discussion by a reviewer (CD) in the form of a definitive statement made by the authors and supported by the presentation of data. Qualitative findings and the quantiative findings presented in narrative form were pooled. Findings were first inductively grouped into categories that were created on the basis of similarity of meaning; categories were then subjected to a meta-aggregation in order to produce a single comprehensive set of synthesized findings that could be used as a basis for evidence-based practice which would inform policy makers and practitioners on the facilitators and barriers associated with implementing a CCM [23].

## Results

### Description of studies

**Fig. 1:**
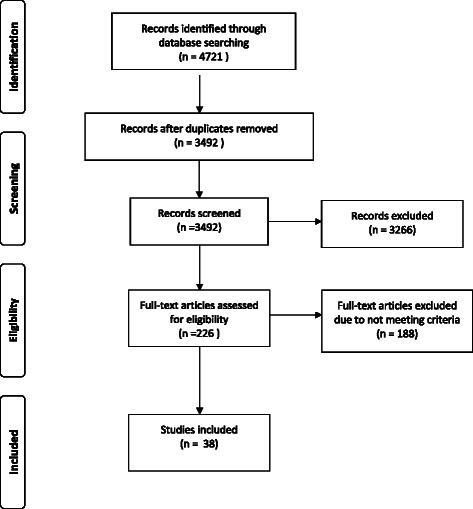
Summary of literature search

The search of information sources returned 3492 articles. The majority of these studies were subsequently excluded based on their title or abstract because they clearly did not meet the inclusion criteria for this review. A total of 226 full text articles were obtained and a further 188 were excluded as they did not meet the criteria. Thirty eight published peer-reviewed articles were ultimately included in this review (Fig. 1).

The majority of studies were conducted in the Americas, including United States of America, Canada and Mexico [24–47]. Nine studies were also conducted in Europe including United Kingdom, Spain, Belgium, Italy, Denmark, Netherlands and Germany [48–56]. Four studies were conducted in Australia and New Zealand [57–60] and one study in Africa [61].

While all studies described in the included papers were conducted within a primary healthcare setting, the majority focused on the provision of care for diabetes [24, 26, 28–30, 32–34, 36, 37, 39, 40, 42, 45, 47, 49, 53–55, 60, 61]. Included studies also focused on cardiovascular disease [28, 48, 60], depression [32] and chronic obstructive pulmonary disease [50, 52, 60]. Other studies [25, 27, 31, 35, 38, 41, 43, 44, 46, 51, 56–59] focused on the provision of care to patients with chronic diseases more generally.

Though a range of CCM elements were used across the papers reviewed, the mean number of elements across the 38 papers included in this review was four, with only one study including seven of the elements. None of the papers included studies utilising FS. While the most commonly included element was SMS (Table 1), there were substantive between study variations both in the elements used and how these elements were implemented. For example, descriptions of SMS implemented in primary care settings included development of care guides and individualised patient action plans [36, 48] individual counselling or coaching [52, 54], education programs on disease management [29, 32, 39, 50], web-based patient portals [30] and programs on empowerment, goal-setting and motivation [35]. More generally, a number of papers in this review reported using plan-do-study-act or learning collaborative approaches which resulted in context specific implementation strategies for all included elements [24, 33, 35, 42, 60] (Table 1).

**Table 1:** Overview of CCM Elements Reviewed

Element	Number of Papers
Self-Management Support [SMS]	31
Delivery System Design [DSD]	27
Decision Support [DS]	26
Clinical Information Systems [CIS]	25
Health Organisational Change [OC]	10
Case Management [CM]	9
Community Support [CS]	9
Family Support [FS]	0

### Methodological quality

All 38 papers were critically appraised. The Cochrane Collaboration’s tool was used to assess risk of bias in randomised controlled trials, non-RCT quantitative studies, non-RCT qualitative studies and mixed-methods evaluations [21]. Case studies and case series were assessed in accordance with the Joanna Briggs Reviewers’ Manual [23]. Additional files present the results of the appraisal process applied to all studies [Additional files 3, 4, 5, 6 and 7].

### Facilitators and barriers

The objective of this review was to identify the facilitators and barriers to implementing chronic care models. Of the 38 papers included in this review, four reported on randomised control trials [25, 32, 47, 51], three on cohort studies [26, 53, 54], two on cross sectional studies [28, 59], 11 on qualitative studies [27, 30, 31, 34, 41, 44, 45, 49, 54, 56, 57] and 17 case studies or case series [24, 29, 33, 35–40, 42, 43, 46, 48, 50, 58, 60, 61]. All findings related to identifying facilitators and barriers to implementing chronic care models regardless of perspective, disease or geographical locations were pooled to generate one cohesive set of synthesized findings (Table 2). As such, the syntheses represent provider and provide perspectives.

**Table 2:** Summary of Synthesised Findings

Synthesised Finding 1 - Acceptability of CCM interventions
• Category: Acceptability of the CCM intervention for healthcare providers
• Category: Acceptability of CCM interventions for patients
Synthesised Finding 2 - Preparing healthcare providers for a CCM
• Category: Information about the change
• Category: A reason to change
• Category: Appropriately qualified and experienced chronic care staff
• Category: Leaders and champions for success
Synthesised Finding 3 - Supporting patients
• Category: Patients supported and encouraged to engage with care
• Category: Acknowledging patient differences
Synthesised Finding 4 - Resources for implementation and sustainability
• Category: Time needed to implement and sustain CCMs
• Category: Information and communication
• Category: Sufficient funding
• Category: Collaborations with other healthcare services
• Category: Monitoring and evaluating

From the 38 included papers, findings pertaining to both facilitators and barriers to the implementation of CCMs in a primary care setting were extracted. Qualitative as well as quantitative findings presented in a narrative form were grouped into ten categories and which were then meta-aggregated into four synthesized findings.

### Synthesised finding 1 – acceptability of CCM interventions

One of the most prominently reported factors influencing the successful implementation of CCMs was acceptability. Generally referred to using terms such as “satisfaction”, 15 of the 38 papers included in this review reported on acceptability from the perspective of healthcare providers [24, 36, 43, 52], patients [25, 30, 32, 38, 49, 58] or both providers and patients [35, 37, 41, 48, 53]. The majority of these participants felt that the CCM implemented in their setting was acceptable.

#### Category: Acceptability of the CCM intervention for healthcare providers

The majority of papers considered acceptability from the point of view of the healthcare providers [24, 35, 36, 48, 53]. These papers report high levels of support for CCM elements, which in turn facilitated their implementation. Not all, however, provided reasons for why healthcare providers felt these CCM elements were acceptable. Those that did suggested that healthcare providers found them to be helpful to their work [24] and perhaps more importantly, believed that they would make a positive impact on their patients’ health [48]. One paper also reported that healthcare providers experienced greater work satisfaction and had access to additional resources as a result of the model’s implementation [35]. Finally, one paper focused on the acceptability of the training used to prepare staff for implementation, rather than focusing on implementation of CCM per se [43].

#### Category: Acceptability of CCM interventions for patients

Of the studies which did measure patients’ perspectives, the majority found that CCMs were acceptable [35, 37, 38, 49, 51, 53]. Nevertheless, two RCTs found no statistically significant differences in levels of satisfaction between intervention and control patients [25, 32]. Another qualitative study identified a range of both positive and negative responses in relation to a study which aimed to provide patients with online information as part of SMS [30]. Positive responses in this study included patients feeling empowered as a result of the readily available online information, as well as a greater understanding of how lifestyle choices impacted upon their health. On the other hand, patients in this study also reported a number of inefficiencies which reduced the acceptability of the system, including missing online results and slow response times from nurses and doctors.

### Synthesised finding 2 - preparing healthcare providers for a CCM

Factors which influenced whether healthcare providers embraced the implementation of a CCM also depended on whether sufficient information was provided in an appropriate manner and whether staff were convinced that a change to the way healthcare was delivered would be beneficial. This synthesised finding acknowledged that without staff who had the necessary skills and experience to take on new roles and responsibilities, implementing a new CCM would be particularly difficult. Also noted, was the importance of ensuring that healthcare staff are supported by strong leaders and champions who are able to provide both management and clinical support.

#### Category: Information about the change

Clearly articulated concepts and examples of how a CCM could work once implemented, were identified as an important facilitator to implementation [28]. Staff who were not provided with this information may be left wondering what the expected outcomes could or should be [32]. Structured learning sessions involving a whole of team approached that focused on collaborative and supportive learning environments, providing opportunities for staff to ask questions and raise concerns, were thought to prevent any resistance to change [26, 34].

Ensuring that individual staff members have the necessary knowledge and skills required to undertake their particular roles and manage any new responsibilities prior to implementing a new CCM was also shown to be important [59]. If, for example, the model included community or family support, it could be particularly advantageous for staff to know about community resources including existing disease management group meetings, exercise facilities, mental health services, or discounted health programs [35]. It was also considered important for staff to feel comfortable and confident in taking on any new responsibilities; if necessary by being provided with opportunities for additional training and on the job support [61]. In order to facilitate fruitful working relationships, staff who needed to collaborate with people external to their immediate team or an external organisation were believed to have benefited from being provided with information about and even a chance to meet with these collaborating parties prior to implementation [54].

#### Category: A reason to change

One of the most important facilitators to implementing a CCM is a well thought out and articulated argument for change [60]. Without clearly defined benefits, healthcare providers may become dismissive and uncooperative. A groundswell of agreement for improvements needs to be carefully nurtured prior to beginning the implementation process [31]. Quality Improvement initiatives that clearly identify gaps in care [44], where the goal can be clearly recognised as improvements to patient care rather than change for the change sake of change were considered to be a useful strategy [56]. Goals and outcomes that appear unclear or fuzzy, and a process of change that were uncoordinated, were believed to result in healthcare providers disengaging from the implementation process [31]. Managers, therefore, played an important role in leading staff through the change process, which was further enhanced by ensuring that any success was measured and appropriately rewarded [41].

#### Category: Appropriately qualified and experienced chronic care staff

Unsuitable or insufficient staffing undermined the implementation and sustainability of a CCM [27]. While physicians were considered to be an essential component of the chronic care team particularly in regards to advising and supporting other healthcare providers [61], the lack of nurses dedicated to chronic disease programs [61], as well as management and administrative support staff [24, 31, 55], impeded the implementation and/or sustainability of a new CCM.

A high turnover of staff was noted as another barrier to both implementing and sustaining a new CCM [61]. In one instance [26] a general shortage of qualified healthcare providers meant that highly skilled staff were being replaced by less adept medical assistants which in turn put at risk the sustainability of the CCM. High staff turnover, in this instance, resulted in a complete derailing of the implementation process [33]. Irregular rotations of both doctors and nurses in another remote location created a lack of consistent chronic disease care, which was vital to the success of a given model [59]. Yet on a more positive note, the implementation of a new CCM in one study [41] was believed to be associated with a decrease in staff turnover.

Skills and experiences of chronic care staff were also important for the success of a new CCM. Although providing staff had a desire to learn, and sufficient time to devote to understanding new ways of working, shortfalls in any skills or experience could be overcome [28]. Another way of supporting staff through the change process was to form multi-disciplinary teams [42]. Yet, setting up a multidisciplinary team was not always easy. Respect for the role of each discipline and enhanced interdisciplinary communication were critical to the success of this initiative [54]. Furthermore, if existing staff had no prior history of working within an interdisciplinary team the sustainability of the model may be put at risk [54].

#### Category: Leaders and champions for success

A consistent theme within the papers reporting upon facilitators and barriers was the need for supportive leadership [24, 26, 31, 34, 35, 41, 42, 60]. As well as management staff who were committed to the implementation and sustainability of the new model [24, 42], strong clinical leaders and champions were needed to support healthcare providers through the change process [31]. In a primary care clinic within a teaching hospital physician leaders were found to be essential in helping a provider population of rotating residents and part-time physicians implement a CCM model. Indeed the educationally rich environment fostered by these leaders was felt to benefit temporary and permanent staff members alike [26]. Without this type of support, the implementation and sustainability of the model may be put at risk [31, 34].

### Synthesized finding 3 - supporting patients

The third synthesised finding identified factors that were believed to influence whether patients were able and willing to engage with care delivered through a CCM. In particular, patients needed to be supported to fully engage with healthcare, particularly when a model incorporated aspects of self-management support. Providing understandable information about their health, as well as support groups that motivated them to reach their own goals, encouraged patients to take a greater interest in and responsibility for their health. This finding also identified that patients may not always be able to actively contribute to their care. Instead, it was important to acknowledge patients as unique individuals with different levels of capacity for engagement.

#### Category: Patients supported and encouraged to engage with care

Self-management support, which relied on patients taking some responsibility for their own healthcare, was one of the most common elements identified in this review (see Description of Studies). Educational services that provided clear and concise information to patients so that they were able to respond appropriately were generally viewed positively [45, 54]. Yet educating and empowering patients was a challenge given the breadth of clinical questions that may need to be covered, the nature of patients’ concerns and anxieties, patients’ varying cultural needs, and related difficulties of concordance and adherence [53]. Support groups were another way of encouraging patients to take on a degree of responsibility for their own care. Support groups were found to be mutually motivating and patients participating in such groups were found to monitor their condition more closely and respond to health promoting activities such as physical exercise, more positively [47]. Support groups were often seen as a particularly beneficial adjunct to general healthcare.

However, not all patients were ready or able to take on greater responsibility for their own healthcare [58]. In particular, poor psychological health (health beliefs, motivation and self-efficacy), lower levels of education (poor knowledge or awareness of education services), and other social determinants of health (finance, transport), as well as psychosocial factors (discrimination due to having diabetes, lack of support from family, friends or the community and inappropriate cultural messages), can all act as major barriers to diabetes care [40]. Other interventions including online systems that allowed patients to monitor their own records did not suit all patients, especially if many of the target group did not have the necessary skills to navigate these sometimes complex systems [56].

#### Category: Acknowledging patient differences

Another barrier to implementing self-management support was that advice provided in educational activities was not personalised to the individual patient [47]. A client- or patient-centred approach was considered to be far more effective in supporting patients to take responsibility for their own health [35, 57]. Individualised self-management plans with dedicated time to speak to clients in order to ensure they have all of the relevant information and ability to implement the plan is required [59]. However, not all healthcare facilities were set up to provide this level of care. Walk-in clinics may not have the time and solo family practices may not have the staff required to provide extensive patient-centred self-management support [46].

In addition to patient-centred care, there was also a need to ensure that programs were tailored to the needs of the community or region more generally [54]. In particular, language and literacy issues were a challenge to changing delivery system design. Strategies for addressing these included recruiting multilingual staff, adapting and translating materials, redesigning educational handouts towards a pictorial focus, and using interpreters [50].

### Synthesised finding 4 - resources for implementation and sustainability

Features that supported implementation and sustainability more broadly included the time and effort required to implement a new CCM, as well as the need for sufficient resources, including information and communication systems and funding. Ongoing monitoring and evaluation to ensure continuous quality improvements was then needed to ensure the sustainability of CCMs.

#### Category: Time needed to implement and sustain chronic care models

Key to implementation was the need to maintain realistic expectations regarding the time required to implement a CCM [31]. While people may have wanted or wished that changes were quickly realised, in reality it took time for healthcare providers and patients to come to trust the new initiative [54]. Attempting to make too many simultaneous changes to existing delivery of care practices could also discourage staff from moving towards a new model of care [31]. Instead, introducing the model slowly and carefully, with sufficient time for the necessary cultural shifts as the healthcare team take on new roles and responsibilities, was believed to be important for success [58].

Even once implemented, new ways of delivering services appeared to require more, rather than less, staff time [24]. One study [57] found that the amount of time required to conduct patient-centred care planning was a serious barrier to implementing their CCM more widely. Even when supposedly time saving devises such as electronic medical information systems were implemented health providers found that such initiatives took a significant amount of effort to integrate these into their daily practice [45, 56]. Motivating patients to participate in education programs [54], developing patient treatment plans, encouraging self-management and meeting preventive and psychosocial needs of chronically ill patients [41], were all found to require additional healthcare provider time, which should be recognised and factored into daily work schedules.

#### Category: Information and communication

Appropriate information and communication systems were considered to be vital tools for the implementation and sustainability of a new CCM. These systems assisted by identifying and keeping track of patients with chronic disease [42, 58], monitoring healthcare against service standards, identifying gaps in services, and documenting successes [29]. Information and communication systems also aided in self-management support, for example, by using a patient portal to connect with clients and providing up to date information on their health as well as tips for continuing to reduce their risk of further complications from their chronic disease [26, 56].

Nevertheless, information and communication systems that were inappropriately designed or did not function well were a barrier to the implementation and sustainability of CCMs. Healthcare providers were critical of, for example, systems that simply replicated existing manual systems, electronic health records that were limited in terms of not being able to provide reminders in real time, and electronic records that required a significant amount of time to enter or retrieve information [56, 53]. In addition, the simultaneous demands associated with the implementation of a electronic medical record system while at the same time changing the way in which care is delivered were thought to be overly onerous [31] . Intensive support was needed to ensure that information and communication systems facilitated rather than hindered the implementation and sustainability of a new CCM [44].

#### Category: Sufficient funding

The implementation and ongoing sustainability of CCMs was sometimes costly, and without sufficient funding, the process was likely to fail [54]. Unfortunately, healthcare services often found it difficult to find the funds to support clinical change, especially when there were other projects competing for the same pot of money [41]. In particular, specialised services such as support groups, which are generally seen as a facilitator to implementation, could require significant amounts of money to fund [47]. Funding some of the basic services such as case management and care planning meetings, important elements to many of the CCMs discussed in this review, were also beyond the budget of some organisations [57]. Yet, incentivising healthcare providers to improve healthcare practices, in combination with the implementing a CCM [27], and possibly even a separate reimbursement for follow-up care or performance-based pay, increased the use of CCMs in practice [32].

On the positive side one study [35] found that increased visits for patients as a direct result of the implementation of a CCM provided additional income to offset any initial loss of revenue. Likewise, another study [39] implemented new patient scheduling arrangements to ensure provider productivity and cost effectiveness for Shared Medical Appointments.

#### Category: Collaborations with other healthcare services

Partnering with other healthcare services such as hospitals and specialist services was considered to facilitate the implementation and sustainability of CCMs. In particular, collaboration was linked with cross institutional learning [42] and communication [53], joint decision making [54, 60], pooling of scarce resources [34, 62]. Other important features of collaborations was the access to healthcare services which otherwise may not have been available [45], and improved transitioning of patients between healthcare services [43].

#### Category: Monitoring and evaluating

Finally, CCMs required systems for ongoing monitoring and evaluation if they were to be effectively implemented and sustained [27, 59]. One of the primary barriers to the process of continuous quality improvements is the lack of useful data and poor collection of existing measures [26, 31]. Yet a system for monitoring and evaluation was a hindrance if providers perceived that it did not add particular value but instead was an additional burden [54].

## Discussion

This systematic literature aimed to identify facilitators and barriers to implementing a CCM in a primary healthcare setting from the perspectives of healthcare providers and patients. The four synthesised findings – Acceptability of the CCM intervention, Preparing Healthcare Providers for the CCM, Supporting Patients, and Resourcing Implementation and Sustainability – spoke to a need to consider an holistic approach to CCM implementation and sustainability both from patients’ and healthcare providers’ perspectives. While it is important to consider whether the healthcare system will be able to support the implementation of a CCM, this review highlighted the importance of human factors to the success or otherwise of CCM interventions [62].

### Facilitators and barriers

Whether or not the CCM was acceptable to both patients and providers was a factor for determining the success of the interventions included in this review. However, definitions of acceptability varied. One of the primary difficulties in measuring acceptability is that the term is often inclusive of a number of different constructs including whether the patient is willing to implement changes to their behaviour [63]. Early work in this field suggests that from a patient’s perspective, these constructs can include social validity, which refers to the social desirability of an intervention [64]. In addition, concepts such as treatment integrity and treatment use [65] have also been used in to better understand whether individuals like a prescribed treatment or procedure [66]. Adding to this complexity is the number of underlying issues that influence the degree to which any individual finds an intervention acceptable. For patients this may include the severity of their condition [67] and the quality and amount of information that is available to them [68]. The reputation of the service, the number of alternative healthcare options and previous experiences also influence patients’ perceptions [69]. Very few studies, however, considered acceptability from a healthcare provider perspective. In addition, simply asking whether a patient or healthcare provider liked or was satisfied with a particular intervention may therefore not be a reliable method for measuring this construct.

The papers included this review also suggested that preparing healthcare providers for change was an important factor for success. If the information provided is not sufficient, or alternatively if healthcare providers do not see the benefits of implementing a CCM, it is more likely to fail. This highlights the importance of leaders and champions for guiding their healthcare staff through the change process. These are the people who not only sell the vision for the future but also legitimise the change and “call people to action” ([70] p. 366). Effective leaders will involve their staff from the very beginning of the change process to help embed a sense of ownership [71].

Patients must not be left to fend for themselves but instead should receive support as part of the intervention. Yet none of the studies described in this review utilised FS, and only nine of the papers utilised CS. However, the review did find that it was important to appreciate patients’ individual capacity to respond to self-management support initiatives. Not only the degree of support, but also the type of support needed, may vary across time and therefore healthcare providers will need to continually monitor patient needs. Importantly, a team approach, whereby a range of healthcare providers are available to a patient at any one point in time, may best support patients’ needs [72]. Other important factors that influence the success of self-management initiatives include ensuring that patients are able to access appropriate levels of information in a format that they are able to understand, identifying whether patients have the desire and resources to manage their own health, being able to help patients plan strategies that contribute to their particular goals and ensuring there is mutual investment, with both the healthcare provider and the patient working towards common goals [73].

This systematic literature review also identified the importance of ensuring appropriate resources are in place to support change. Many of the CCM elements including case management and self-management support require healthcare providers to spend more, not less, time with patients [74]. Yet insufficient funding for employing additional chronic care staff as well as issues pertaining to recruiting and retaining healthcare providers particularly in rural and remote areas [75] often means that time for patients is at a premium. The time needed to develop and use a clinical information system was also highlighted. The perceived ease of use is also an important acceptance criteria for whether a new technology will be accepted and used by healthcare providers [76].

### A greater focus on the human factors

Three of the four synthesised findings in this systematic literature review highlighted the significant contribution that patients and providers can make in either facilitating or impeding the implementation of CCMs. However, even the crucial resources identified in the fourth synthesised finding such as time, underlined the importance of human factors for implementation and sustainability. Obstacles to implementation may therefore be as much about the people involved, as they are about resources, processes and systems. Yet, the two theories that are thought to inform the development and underpin the philosophy behind CCMs – Integrated Care and Quality Management – have tended to take a more structural or systems approach to the delivery of care [9].

Although not always clearly defined, the concept of Integrated Care grew from the notion that the development of “coherent set of methods and models on the funding, administrative, organisational, service delivery and clinical levels” ([77], p. 3) will lead to better connectivity between healthcare services. More recently, Integrated Care has evolved to become more synonymous with individual patients’ needs [78]. Some researchers [79, 80] going so far as to call for the development of evaluation measures and techniques which capture broader and more nuanced understandings of patient perspectives. Generally, there is a move away from regarding patients as passive recipients of healthcare to one which acknowledges their active participation in making choices about the way in which their health is managed [81].

Quality management theory also started out by emphasising the organisational level perspective [82]. This theory originated from the manufacturing sector where quality was first assured through the inspection of products prior to despatch. Quality control which aimed to find defects during the production process, quality assurance which developed processes that prevented defects and finally total quality management which utilised a management approach to ensuring an entire quality system, have also been developed [10]. Within healthcare, quality management theory has tended to focus on the total quality management approach, seeking to design and control systems in order to minimise harm to patients [8]. Yet more recently there is a recognition that commitment to improving services from healthcare providers is crucial to the success of quality initiatives [83]. Rather than thinking about quality at just the system level, “quality systems that give staff ongoing “ownership” and pride in a way that is akin to the era of the craftsmen” ([84], p. 367) has been called for. As was found in this review, commitment and support from leaders is particularly crucial for the successful implementation of quality management programs in healthcare settings.

This systematic literature review therefore mirrors the more recent progression in thinking behind both Integrated Care and Quality Management theories by re-emphasising the human factors which need to be considered when implementing complex interventions such as CCMs. While others have suggested that the implementation of complex intervention primarily depends on the behaviour of healthcare providers , this review suggests that patients can also act to facilitate or impede the implementation of CCMs.

#### Limitations

While no papers were excluded based on quality, of particular concern was the risk of bias, particularly in the case of one author (CD) being responsible for the data extraction. In addition, the quality of the case studies and case series included in this review was considered to be poor. Yet the findings from this systematic literature review are supported by more recent shifts in two of the primary theories – Integrated Care and Quality Management – which have informed the development of CCMs. It is important to acknowledged that the vast majority of included studies were conducted in the Americas. While US, Canadian and to some extent Mexican perspectives are well represented, the results may not thoroughly reflect facilitators and barriers to intervention implementation in the other countries. The authors also acknowledge that to be included in this review the paper had to have reported on an intervention which included at least two of the eight specified elements (CS, FS, CM, SMS, OC, DSD, DS, CS). It is probable that there will be other CCMs which do not include two of these elements. Finally, the authors also acknowledge that the key findings may be very different had papers reporting the perspectives of other stakeholders including, for example, policy makers been sought.

## Conclusion

The successful implementation of complex interventions such as a CCM may depend not only on the provision of appropriate resources and the development of effective systems and processes, but also on a broad range of different stakeholders who will interpret and influence this implementation process. This systematic literature review has re-emphasised the need to consider the human factors, including the role of both patients and healthcare providers, who can either facilitate or impede successful implementation. In addition to ensuring appropriate resources, this review highlights the importance of ensuring that the intervention is acceptabile to both patients and healthcare providers. It was also emphasises the impotance of preparing healthcare providers for the change process and ensuring that patients are supported throughout the implementation of a CCM.

## Additional files

Additional file 1: **Medline Search Strategy.** (DOCX 13 kb)
Additional file 2: **Description of Chronic Care Models.** (DOCX 19 kb)
Additional file 3 **Quality appraisal of Case studies and Case series.** (DOCX 44 kb)
Additional file 4: **Non-Randomised Control Trials.** (DOCX 14 kb)
Additional file 5: **Appraisal for qualitative research.** (DOCX 43 kb)
Additional file 6: **Risk of bias in randomised controlled trials.** (DOCX 15 kb)
Additional file 7: **Retrospective Studies.** (DOCX 15 kb)

## Abbreviations

CCM: Chronic Care Model
CIS: Clinical information systems
CM: Case management
CS: Community support
DS: Decision support
DSD: Delivery system design
FS: Family support
OC: Health organisational change
SMS: Self-management support
