# Supplementary material for: Factors influencing the implementation of chronic care models: A systematic literature review
Source: BMC Fam Pract. 2015 Aug 19;16:102. doi: 10.1186/s12875-015-0319-5 (PMC4545323; doi:10.1186/s12875-015-0319-5)
Supplement: Additional file 1: — Medline Search Strategy. (DOCX 13 kb) [file 12875_2015_319_MOESM1_ESM.docx]

**Medline Search Strategy**

1. "Chronic care model*".tw.

2. (Chronic care adj5 model*).tw.

3. (Model* adj5 (collaborative adj5 care)).tw.

4. (Chronic care adj5 framework*).tw.

5. (Chronic disease adj5 care).tw.

6. (Chronic illness adj5 care).tw.

7. (Model* adj2 care).tw.

8. (Wagner* adj5 model*).tw.

9. (Wagner* adj5 chronic care model*).tw.

10. (Flinder* adj5 model).tw.

11. (Stanford* adj5 model).tw.

12. "delivery of health care"/ or "delivery of health care, integrated"/

13. models, organizational/

14. patient care management/

15. (Model* adj3 care).tw.

16. 1 or 2 or 3 or 4 or 5 or 6 or 7 or 8 or 9 or 10 or 11 or 12 or 13 or 14 or 15

17. "Primary health care".mp.

18. "Primary care".tw.

19. (Primary health adj5 service).tw.

20. exp General Practice/

21. comprehensive health care/ or exp primary health care/

22. community health services/ or community health nursing/ or community mental health services/

23. preventive health services/ or exp health education/

24. 17 or 18 or 19 or 20 or 21 or 22 or 23

25. Chronic Disease/

26. cardiovascular diseases/ or cardiovascular abnormalities/ or cardiovascular infections/ or heart diseases/ or vascular diseases/

27. renal insufficiency, chronic/ or exp kidney failure, chronic/

28. chronic kidney disease.tw.

29. heart disease*.tw.

30. cardiovascular disease*.tw.

31. exp Pulmonary Disease, Chronic Obstructive/

32. chronic respiratory disease*.tw.

33. exp Diabetes Mellitus, Type 2/

34. (diabetes adj5 care).tw.

35. Depression/

36. depression.tw.

37. (depressive or dysthym*).mp.

38. depressive disorder/ or depressive disorder, major/ or dysthymic disorder/

39. exp HIV/

40. 25 or 26 or 27 or 28 or 29 or 30 or 31 or 32 or 33 or 34 or 35 or 36 or 37 or 38 or 39

41. 16 and 24 and 40

42. limit 41 to (humans and yr="1998 -Current")
