# Supplementary material for: Factors influencing the implementation of chronic care models: A systematic literature review
Source: BMC Fam Pract. 2015 Aug 19;16:102. doi: 10.1186/s12875-015-0319-5 (PMC4545323; doi:10.1186/s12875-015-0319-5)
Supplement: Additional file 2: — Description of Chronic Care Models. (DOCX 19 kb) [file 12875_2015_319_MOESM2_ESM.docx]

**Description of Chronic Care Models**

| **First Author**  **(Year)** | **Geographical Context** | **Study Type** | **Chronic Disease Focus** |
| --- | --- | --- | --- |
| Barcelo (2010) | Central and South America | RCT | Diabetes |
| Chin (2004) | USA | Case Studies & Series | Diabetes |
| Ciccone (2010) | Europe | Case Studies & Series | CVD |
| Coleman (1999) | USA | RCT | Chronic Disease |
| DiPiero (2008) | USA | Cohort Studies | Diabetes |
| Feifer (2001) | USA | Cross Section | Diabetes, CVD |
| Feifer (2006) | USA | Qualitative | Chronic Disease |
| Friedman (1998) | USA | Case Studies & Series | Diabetes |
| Fuller (2004) | Australia | Qualitative | Chronic Disease |
| Green (2006) | Canada | Qualitative | Chronic Disease |
| Hess (2007) | USA | Qualitative | Diabetes |
| Hroscikoski (2006) | USA | Qualitative | Chronic Disease |
| Johnson (2006) | Europe | Qualitative | Diabetes |
| Katz (2009) | Africa | Case Studies & Series | Diabetes, Other Disease Focus |
| Landis (2006) | USA | Case Studies & Series | Diabetes |
| Landis (2007) | USA | RCT | Diabetes, Depression, Other Disease Focus |
| Lemay (2010) | USA | Qualitative | Diabetes |
| Lemmens (2009) | Europe | Case Studies & Series | COP |
| Lyon (2011) | USA | Case Studies & Series | Chronic Disease |
| McCulloch (1998) | USA | Case Studies & Series | Diabetes |
| McCulloch (2000) | USA | Case Studies & Series | Diabetes |
| Martin (2012) | Europe | RCT | Chronic Disease |
| Martin (2008) | Australia | Case Studies & Series | Chronic Disease |
| Meulepas (2007) | Europe | Non RCT | COP |
| Mohiddin (2006) | Europe | Cohort Studies | Diabetes |
| Nasmith (2004) | Canada | Qualitative | Diabetes |
| Reuben (2011) | USA | Case Studies & Series | Chronic Disease |
| Rondeau (2009) | Canada | Case Studies & Series | Chronic Disease |
| Sanchez (2011) | USA | Case Studies & Series | Diabetes |
| Si (2008) | Australia | Cross Section | Chronic Disease |
| Siminerio (2005) | USA | Case Studies & Series | Diabetes |
| Sunaert (2010) | Europe | Cohort Studies | Diabetes |
| Sunaert (2009) | Europe | Qualitative | Diabetes |
| Wagner (1999) | USA | Qualitative | Chronic Disease |
| Walters (2012) | Europe | Qualitative | Chronic Disease |
| Wang (2004) | USA | Case Studies & Series | Diabetes |
| Weinstein (2011) | USA | Case Studies & Series | Chronic Disease, Other Disease Focus |
| Wellingham (2002) | Canada | Case Studies & Series | Diabetes, CVD, COP, Other Disease Focus |
