# Supplementary material for: Factors influencing the implementation of chronic care models: A systematic literature review
Source: BMC Fam Pract. 2015 Aug 19;16:102. doi: 10.1186/s12875-015-0319-5 (PMC4545323; doi:10.1186/s12875-015-0319-5)
Supplement: Additional file 3 — Quality appraisal of Case studies and Case series. (DOCX 44 kb) [file 12875_2015_319_MOESM3_ESM.docx]

# **Quality appraisal of Case studies and Case series**

| **AUTHOR/DATE** | **Q1*** | **Q2*** | **Q3*** | **Q4*** | **Q5*** | **Q6*** | **Q7*** | **Q8*** | **Q9*** |
| --- | --- | --- | --- | --- | --- | --- | --- | --- | --- |
| Chin (2004) | Yes | Yes | No | Yes | Not applicable | Yes | No | Yes | Yes |
| Ciccone (2010) | No | Yes | No | Yes | Not applicable | Yes | No | Yes | Yes |
| Friedman (1998) | No | No | No | Yes | Not applicable | Yes | No | Unclear | Unclear |
| Katz (2009) | Not applicable | Yes | No | Yes | Not applicable | Yes | No | Yes | Yes |
| Landis (2006) | No | Yes | No | Yes | No | Yes | Not applicable | Unclear | Unclear |
| Lemmens (2009) | No | Yes | No | Yes | Not applicable | Yes | No | Yes | Yes |
| Lyon (2011) | No | Unclear | No | Unclear | Not applicable | Unclear | Not applicable | Unclear | Unclear |
| Martin (2008) | No | No | No | Not applicable | Not applicable | Not applicable | Not applicable | Not applicable | Not applicable |
| McCulloch (1998) | Yes | Yes | No | Yes | Yes | Yes | Not applicable | Yes | Yes |
| McCulloch (2000) | No | Yes | No | Yes | Not applicable | Yes | No | Yes | Unclear |
| Reuben (2011) | No | Not applicable | No | Unclear | Not applicable | Not applicable | Not applicable | Unclear | Unclear |
| Rondeau (2009) | No | Yes | Yes | Yes | Yes | Not applicable | Not applicable | Yes | Yes |
| Sanchez (2011) | Not applicable | Yes | No | Yes | Not applicable | Yes | No | Yes | Yes |
| Siminerio (2005) | No | Yes | No | Yes | Not applicable | Yes | No | Yes | Yes |
| Wang (2004) | Not applicable | Not provided | No | Unclear | Not applicable | Unclear | Not applicable | Unclear | Unclear |
| Weinstein (2011) | Not Applicable | No | None identified | No | Not applicable | Not applicable | Not applicable – staff in one facility only | No – unsure how acceptability of training was measured | Not applicable |
| Wellingham (2003) | Partially | No | No | Unclear | Unclear | Unclear | Unclear | Unclear | Unclear |

* Refer Appendix ???
