# Supplementary material for: Factors influencing the implementation of chronic care models: A systematic literature review
Source: BMC Fam Pract. 2015 Aug 19;16:102. doi: 10.1186/s12875-015-0319-5 (PMC4545323; doi:10.1186/s12875-015-0319-5)
Supplement: Additional file 4: — Non-Randomised Control Trials. (DOCX 14 kb) [file 12875_2015_319_MOESM4_ESM.docx]

Non-Randomised Control Trials

| **AUTHOR/DATE** | **SELECTION BIAS** | **SAMPLING BIAS** | **DETECTION BIAS** | **ATTRITION BIAS** | **REPORTING BIAS** | **OTHER BIAS** |
| --- | --- | --- | --- | --- | --- | --- |
| Meulepas (2007) | High risk: randomisation based on regional distribution. | Unclear: insufficient information about how intervention practises were chosen over the wait-list (control) practises. | Unclear: lab assistant blinded? | High risk: high attrition rate. Drop-outs from baseline measurements kept in analysis to improve power. | Low risk: outcomes clearly reported. | N/A |
