# Supplementary material for: Factors influencing the implementation of chronic care models: A systematic literature review
Source: BMC Fam Pract. 2015 Aug 19;16:102. doi: 10.1186/s12875-015-0319-5 (PMC4545323; doi:10.1186/s12875-015-0319-5)
Supplement: Additional file 5: — Appraisal for qualitative research. (DOCX 43 kb) [file 12875_2015_319_MOESM5_ESM.docx]

# Appraisal for qualitative research

| **AUTHOR/DATE** | **Q1** | **Q2** | **Q3** | **Q4** | **Q5** | **Q6** | **Q7** | **Q8** | **Q9** | **Q10** |
| --- | --- | --- | --- | --- | --- | --- | --- | --- | --- | --- |
| Feifer (2006) | No | Unclear | Unclear | Unclear | Unclear | No | No | Yes | Unclear | Yes |
| Feifer (2001) | High risk: limited information provided for characteristics of practice group - no patient characteristics or number provided | Low risk: HC centres randomised sample from another study. | Unclear: provider self-reported use of CCM. | Low risk: cross-sectional. | Low-risk: all outcomes reported | N/A | Feifer (2001) | High risk: limited information provided for characteristics of practice group - no patient characteristics or number provided | Low risk: HC centres randomised sample from another study. | Unclear: provider self-reported use of CCM. |
| Fuller (2004) | No | Unclear | Unclear | Unclear | Unclear | No | No | Yes | Unclear | Yes |
| Green (2006) | Yes | Yes | Yes | Yes | Yes | Yes | No | Unclear | Unclear | Yes |
| Hess (2007) | Yes | Yes | Yes | Ues | Yes | No | No | No | Unclear | Yes |
| Hroscikoski (2006) | No | Unclear | Unclear | Unclear | Unclear | Yes | Yes | No | Unclear | Yes |
| Johnson (2006) | No | Unclear | Unclear | Unclear | Unclear | No | No | Yes | Unclear | Yes |
| Lemay (2010) | No | Unclear | Unclear | Unclear | Unclear | No | Yes | Yes | Yes | Yes |
| Nasmith (2004) | No | Unclear | Unclear | Unclear | Unclear | No | No | No | Unclear | Yes |
| Sunaert (2009) | Yes | Yes | Yes | Yes | Yes | No | No | Yes | Yes | Yes |
| Wagner (1999) | No | Unclear | Unclear | Unclear | Unclear | No | No | Yes | Unclear | Yes |
| Walters (2012) | No | Unclear | Unclear | Unclear | Unclear | No | No | Yes | Yes | Yes |
