# Supplementary material for: Factors influencing the implementation of chronic care models: A systematic literature review
Source: BMC Fam Pract. 2015 Aug 19;16:102. doi: 10.1186/s12875-015-0319-5 (PMC4545323; doi:10.1186/s12875-015-0319-5)
Supplement: Additional file 6: — Risk of bias in randomised controlled trials. (DOCX 15 kb) [file 12875_2015_319_MOESM6_ESM.docx]

## Risk of bias in randomised controlled trials

| **AUTHOR/DATE** | **SELECTION BIAS** | **PERFORMANCE BIAS** | **DETECTION BIAS** | **ATTRITION BIAS** | **REPORTING BIAS** | **OTHER BIAS** |
| --- | --- | --- | --- | --- | --- | --- |
| Barcelo (2010) | High risk: patients selected by medical teams based on capacity to communicate, advanced knowledge of diabetes and willingness to collaborate. | High: all clinics selected had implemented clinical information systems. No information provided about random allocation to intervention and usual care | Unclear: no information provided on blinding of assignment or of outcome. | N/A | High: reported ACIC before and after for intervention group only not usual care groups | N/A |
| Coleman (1999) | Low risk: Simple randomisation of physicians | High Risk: open label | High risk:  while some outcome measures e.g. chart reviews were analysed by a blinded reviewer, the other outcomes were not blinded | Low risk: losses to follow up were disclosed and analyses were conducted using a modified intention to treat analysis.  (The overall follow-up rate at 24 months for study population was 89%, (84% for control, 93% for intervention). | Low risk: all pre-specified outcomes were reported. |  |
| Landis (2007) | Low risk: both groups were similar. | High risk: no allocation concealment or blinding. | High risk: no blinding. | Unclear: not reported. | Low risk: outcomes reported. | N/A |
| Martin (2012) | Low risk: clear selection criteria. | Low risk: patients randomised to intervention or control groups. | High-risk: different intervention delivery times. Different follow-up times between centres. | Low risk: low drop-outs. | Low risk: outcomes reported. | Authors have financial interest in DS system tested. |
