# Supplementary material for: Factors influencing the implementation of chronic care models: A systematic literature review
Source: BMC Fam Pract. 2015 Aug 19;16:102. doi: 10.1186/s12875-015-0319-5 (PMC4545323; doi:10.1186/s12875-015-0319-5)
Supplement: Additional file 7: — Retrospective Studies. (DOCX 15 kb) [file 12875_2015_319_MOESM7_ESM.docx]

## Retrospective Studies

| **AUTHOR/DATE** | **SELECTION BIAS** | **SAMPLING BIAS** | **DETECTION BIAS** | **ATTRITION BIAS** | **REPORTING BIAS** | **OTHER BIAS** |
| --- | --- | --- | --- | --- | --- | --- |
| Dipiero (2008) | Low risk: clear description of target practice and population | Low risk: medical record audit, low inclusion threshold. | High risk: control group likely contaminated by CMM initiative implemented in exposure practice. | Low risk: retrospective chart review. | Low risk: outcomes well reported. | N/A |
| Mohiddin (2006) | Unclear: two geographical Primary care groups compared- unknown homogeneity of systems and resources. | High risk: No baseline characteristics reported. | High risk: exposure unclear, concurrent national quality improvement initiative probably contaminated control group. | Low risk: good clinical record recovery. Although low survey response rates (acceptability outcomes). | Low risk: outcomes clearly reported | N/A |
| Sunaert (2010) | High risk: selection of patient cohort based on pre-selected criteria and from laboratories providing patient data. No randomisation or matching. | High risk: quasi-experimental design involving a comparable control region (two different regions). | Unclear risk: indirect criteria used to select patients from six labs in intervention and four labs in control. | Low risk: retrospective cohort. | Unclear risk: | N/A |
